# Illness representation of COVID-19 affected public’s support and anticipated panic regarding the living with the virus policy: a cross-sectional study in a Chinese general population

**DOI:** 10.3389/fpubh.2023.1158096

**Published:** 2023-09-04

**Authors:** Yanqiu Yu, Rachel Hau Yin Ling, Joyce Hoi-Yuk Ng, Mason M. C. Lau, Tsun Kwan Mary Ip, Joseph T. F. Lau

**Affiliations:** ^1^Department of Preventive Medicine and Health Education, School of Public Health, Fudan University, Shanghai, China; ^2^Centre for Health Behaviours Research, School of Public Health and Primary Care, The Chinese University of Hong Kong, Shatin, Hong Kong SAR, China; ^3^Zhejiang Provincial Clinical Research Center for Mental Disorders, The Affiliated Wenzhou Kangning Hospital, Wenzhou Medical University, Wenzhou, China; ^4^School of Mental Health, Wenzhou Medical University, Wenzhou, China; ^5^School of Public Health, Zhejiang University, Hangzhou, China

**Keywords:** illness perception, COVID-19, living with the virus policy, cognitive response, emotional response

## Abstract

**Background:**

There is a global trend for countries to adopt the Living with the Virus (LWV) policy regarding COVID-19. Little is known about the public’s supportiveness and emotional responses (e.g., anticipated panic) related to this policy. Such responses may be associated with illness representations of COVID-19 (i.e., how people think and feel about COVID-19). This novel topic was investigated in this study to facilitate policy-making and health communication.

**Methods:**

A random, population-based telephone survey interviewed 500 adults aged ≥18 of the Hong Kong general adult population from March to April 2022.

**Results:**

The prevalence of the public’s support and anticipated panic regarding the LWV policy, which were negatively correlated with each other, was 39.6 and 24.2%, respectively. The illness representation constructs of consequences, timeline, identity, illness concern, and emotional representations were negatively associated with supportiveness and positively associated with anticipated panic regarding the LWV policy. Illness coherence was significantly associated with policy support but not with anticipated panic. The associations between personal control/treatment control and supportiveness/anticipated panic were statistically non-significant. Moderation analyses showed that the above significant associations were invariant between those with and without previous COVID-19 infection.

**Conclusion:**

Policymakers need to be sensitized about the public’s supportive/unsupportive attitude and potential worry (panic) when adopting the LWV policy. Such attitudes/emotional responses may be affected by people’s illness representations of COVID-19. In general, those who found COVID-19 involving a milder nature and less negative emotions would be more supportive and anticipated less panic under the LWV policy.

## 1. Introduction

The COVID-19 pandemic has become a global experience in the past 3 years. Almost all countries in the world were exposed to some COVID-19 control measures, including vaccination, social distancing, compulsory testing, quarantines, and travel restrictions amid the pandemic. Such measures had reduced the number of COVID-19 infections but involved enormous social, economic, and psychological costs. Unemployment was common and excessive global depression during the pandemic had been evident. With the global mitigations of COVID-19 incidence, milder COVID-19 symptoms, the strong need to recover the economy and social life, and public fatigue toward control measures, more and more countries are adopting the Living with the Virus (LWV) policy. The LWV policy, which refers to removing all the restrictions and control measures related to COVID-19 was supported by the rationales that COVID-19 is ineradicable and substantial community immunity had been established through a high vaccination rate and natural immunity. Countries that are characterized by high vaccination rates, availability of effective COVID-19 treatments, emphasis on individual freedom, relatively good medical systems, and sometimes depend on tourism first adopted the LWV policy. As of April 2022, examples included the U.K., Netherlands, Switzerland, Finland, Denmark, Norway, Singapore, Australia, Thailand, South Korea, and Indonesia.

Despite the trend toward adopting the LWV policy, most countries in the world had been maintaining some control measures and were facing the need and challenge to decide whether and/or when to lift such measures and adopt the LWV policy. Although the policy may bring people’s lives back to ‘normal,’ the World Health Organization warned that the growing number of countries adopting the LWV policy may reduce the effectiveness of disease surveillance and increase the number of COVID-19 infections and mutations (1). The new COVID-19 variants are highly infectious although their severity has been attenuated (2, 3). Yet, highly infectious and severely harmful variants might appear in the future. It is uncertain whether the acquired community immunity would then be adequate to control the pandemic. Thus, some people, especially those from countries having lower levels of preparedness (e.g., low vaccination rates), might not support the LWV policy due to worry about infection and have concerns about lifting all the measures in a sudden. The LWV policy is driven by the government. It is important to understand people’s support and emotional responses regarding the LWV policy; such information would facilitate governmental decisions on the timing of adopting the LWV policy and health promotion to reduce mental distress resulting from the removal of control measures. However, such studies are not available.

Perceptions related to the characteristics of a pandemic affected cognitive, behavioral, and emotional responses to the pandemic (4, 5). Illness representation (i.e., illness perceptions) refers to how people think and feel about a disease (6). It involves cognitive and emotional illness representations. The cognitive constructs capture perceptions about the nature of the disease (e.g., severity, control, and timeline) while the emotional constructs refer to potential concerns and negative emotional responses to a disease (6). Illness representations have explained people’s behavioral and emotional responses to various chronic diseases (e.g., diabetes and hypertension), acute conditions (e.g., acute pain), and infectious diseases (e.g., influenza and COVID-19) (7–11).

Illness representations have strong theoretical and empirical support. Theoretically, illness representations constitute a key component of the Common Sense Model of Self-regulation, which postulates that the stimuli caused by a disease would affect cognitive and emotional illness representations, both of which would affect coping and health-related outcomes in parallel (6). Accordingly, illness representations may be associated with people’s support and emotional responses to the LWV policy, which are parts of the cognitive and emotional responses to the COVID-19 pandemic, respectively. Such associations have not been investigated in the literature. Furthermore, the illness representation constructs of COVID-19 were associated with the practice of COVID-19 preventive measures (e.g., social distancing measures and facemask use) (12, 13), the intention of COVID-19 vaccination (14), and emotional distress related to COVID-19 (e.g., worries about COVID-19 and depression) (5, 15). Illness representations hence provide a systematic way to understand perceptions and emotions related to COVID-19.

Several validated scales of illness perceptions have been developed to assess illness representations of various disease conditions among those with and without the disease (11, 16, 17). The Revised Illness Perception Questionnaire (IPQ-R) (11) has eight cognitive constructs and one emotional construct. (1) The timeline acute/chronic construct refers to how long the patient believes the illness would last (i.e., the duration of the illness). (2) The timeline cyclical construct refers to the extent to which the patient perceives the illness would be cyclical. (3) The consequences construct refers to the expected effects and outcomes of the illness. (4) The personal control construct refers to the extent to which the patient believes that the illness could be controlled via personal efforts. (5) The treatment control construct refers to the extent to which the patient believes that the illness could be cured. (6) The illness coherence construct refers to how much the patient understands or comprehends the illness. (7) The identity construct assesses the level of matching between the patient’s symptoms and the disease’s symptoms; it refers to the number of the respondents’ reported symptoms that are believed to be the disease’s symptoms. (8) The cause construct refers to personal ideas about the cause of the illness. (9) The emotional representations construct refers to negative emotional responses to the illness such as fear, anger, and distress. The 11-item Brief Illness Perception Questionnaire (B-IPQ) included all but one (timeline cyclical) construct of the IPQ-R and added the construct of illness concern to the emotional construct, which refers to how much a patient is concerned about the illness (18). Unlike the IPQ-R which requires the participants to report the number of experienced symptoms associated with the illness, the identity construct of the B-IPQ assesses how much the participant experiences (or expects to experience) symptoms that are related to the illness. The 8-item Brief-Illness Perception Questionnaire for COVID-19 (B-IPQ-COVID-19) (9) used in this study was based on the B-IPQ and some modifications were made. Following some previous studies (19, 20), it did not include the 3-item cause construct (which tends to be disease-specific) as the infection is a clearly known cause.

A knowledge gap clearly exists as no studies have looked at the supportiveness and anticipated panic related to the LWV policy, which has significant global implications as all countries need to decide on whether and when to remove the key COVID-19 control measures (i.e., the LWV policy) to resume ‘normal’ life and revitalize their economy. The policy shift, however, is expected to meet both support and objection as COVID-19-related opinions are often, if not always, polarized (21). Understanding the level of the public’s supportiveness may increase the effectiveness of the LWV policy and reduce mental distress at a population level. Objections toward the LWV policy may be due to the worries about losing protection and hence the exposure to a high risk of infection under the LWV policy. According to the CSM, such supportiveness/worries may depend on people’s illness representations of COVID-19. Hence, the understanding of the associations between illness representations and supportiveness/emotional responses is important and may facilitate effective communication between the government and the general public. The findings also support health promotion as illness representations of COVID-19 are malleable through interventions (22, 23).

Importantly, the illness representations of COVID-19 may change (24). For instance, the availability of effective medical treatment and increasing knowledge about COVID-19 may change some illness representations of COVID-19 (25). Accordingly, those ever-contracted COVID-19 may change their illness representations of COVID-19 due to their experience of COVID-19 infection, including a better understanding of COVID-19 (i.e., illness coherence) and perceptions of stronger immunity against and a lower risk of re-infections, resulting in stronger reluctance to comply with COVID-19 control measures (e.g., social distancing and facemask wearing). Therefore, they may be more supportive of the LWV policy and less panicked under the policy than those never-contracted COVID-19. It was hence hypothesized that the history of COVID-19 infection would be a potential moderator between illness representations of COVID-19 and supportiveness/emotional responses regarding the LWV policy.

Given the above, the present study investigated the levels of (a) support toward the LWV policy, (b) anticipated panic under the LWV policy, and (c) the levels of illness representations of COVID-19 in the Hong Kong Chinese adult general population. The associations between the illness representations of COVID-19 and responses to the LWV policy [(a) and (b)] were tested. In addition, it was hypothesized that previous COVID-19 infection status would moderate the associations between the B-IPQ constructs and the two dependent variables (i.e., support and anticipated panic related to the LWV policy).

## 2. Methods

### 2.1. Study design

A random telephone survey was conducted among Chinese adults aged ≥18 years. It was conducted from March 7th to April 19th, 2022, i.e., during a large COVID-19 outbreak (the fifth wave of the pandemic) in Hong Kong. A total of 480,000 household telephone numbers were randomly drawn from the updated landline telephone directories. To cover unlisted telephone numbers, three additional numbers were generated by randomizing the last two digits of each of the randomly selected numbers. The new and old numbers were merged to form the sampling frame. Invalid numbers were replaced by additional numbers. Interviews were made from 5 pm to 10 pm (10 to 15 min) by experienced interviewers to avoid over-sampling non-working individuals. The household member whose birthday was closest to the interview date was invited to join the study. Unanswered telephone calls were given at least three attempts. Unavailable eligible participants were contacted again by appointment. No incentives were given to the participants. Verbal informed consent was obtained from the participants and the ethics approval was obtained from the Survey and Behavioral Research Ethics Committee of the corresponding author’s affiliated institution (No. SBRE-21-0555A).

A total of 500 valid interviews were conducted. The response rate, defined as the number of completed interviews divided by the number of eligible contacts, was 52.2% (i.e., 500 ÷ 957 × 100% = 52.2%).

### 2.2. Measures

#### 2.2.1. Background information

Socio-demographical information and chronic disease conditions (e.g., hypertension, diabetes, chronic pulmonary diseases, heart diseases, cerebrovascular diseases, dementia, liver diseases, tumors; yes or no response options) were collected.

#### 2.2.2. Support toward the LWV policy

The item was “Based on the current local COVID-19 situation, to what extent would you support the LWV policy (e.g., cancelation of policies on social distancing, compulsory facemask use, free COVID-19 testing, travel restriction, and quarantine) to be exercised in Hong Kong?” The five-point response options were recoded into a binary dependent variable [1 = Yes (supportive/strongly supportive) versus 0 = No (neutral/unsupportive/strongly unsupportive)].

#### 2.2.3. Anticipated panic under the LWV policy

Participants were asked whether they agreed with the statement “You would feel panicked under the LWV policy.” The five-point response options were recoded into a binary dependent variable [1 = Yes (agree/strongly agree) versus 0 = No (neutral/disagree/strongly disagree)].

#### 2.2.4. Illness representations of COVID-19

The 8-item Brief-Illness Perception Questionnaire for COVID-19 (B-IPQ-COVID-19) was modified from the B-IPQ to assess participants’ illness representations of COVID-19 (11-point Likert scales) (9); modifications were made by adding a hypothetical scenario of contracting COVID-19 or replacing the term “illness” with “COVID-19″. The eight items were consequence (“In the case of contracting COVID-19, how much does your illness affect your life”; 0 = no affect at all to 10 = severely affects in my life), timeline (“In the case of contracting COVID-19, how long do you think your illness will continue”; 0 = a very short time to 10 = forever), personal control (“In the case of contracting COVID-19, how much control do you feel you have over your illness”; 0 = absolutely no control to 10 = extreme amount of control), treatment control (“In the case of contracting COVID-19, how long do you think your treatment can help your illness”; 0 = not at all to 10 = extremely helpful), identity (“In the case of contracting COVID-19, how much would you experience symptoms from the illness”; 0 = no symptoms at all to 10 = many severe symptoms), illness concern (“In the case of contracting COVID-19, how concerned are you about your illness”; 0 = not at all concerned to 10 = extremely concerned), illness coherence (“how well do you feel you understand COVID-19″; 0 = do not understand at all to 10 = understand very clearly), and emotional representation [“In the case of contracting COVID-19, how much does your illness affect you emotionally (e.g., does it make you angry, scared, upset or depressed)”; 0 = not at all affected emotionally to 10 = extremely affected emotionally]. The B-IPQ-COVID-19 has been used in several studies investigating cognitive responses to COVID-19 (9, 12).

#### 2.2.5. COVID-19 ever infection

The item was “Have you ever been tested COVID-19 positive by using Nucleic Acid Amplification Testing (NAAT) or Rapid Antigen Testing (RAT)? (yes or no response options).”

### 2.3. Statistical analysis

Descriptive statistics were presented [n (%) for categorical variables and mean (standard deviation) for continuous variables]. Between-group comparisons in scores of the illness representations of COVID-19 by background factors of sex, age group, educational level, and chronic disease status were conducted; independent sample t-test and ANOVA were used, with Cohen’s *d* and eta squared demonstrating the effect size, respectively. Univariable and multivariable logistic regression analyses (adjusted for background factors) were used to test the individual associations between the eight domains of illness representations and the two binary dependent variables (support toward the LWV policy and anticipated panic under the LWV policy). The moderation effects of previous COVID-19 infection status on the association between the illness representations of COVID-19 and the two dependent variables were tested by fitting two sets of models. The first set contained only the main effects (i.e., COVID-19 ever infection status plus one of the eight domains of illness representations) adjusting for the background factors. The second set added the interaction term (e.g., COVID-19 infection × one domain of illness representations of COVID-19) to the corresponding main-effect-only model. Statistical analyses were conducted by using SPSS 23.0. Statistical significance was defined as by two-sided value of *p* < 0.05.

## 3. Results

### 3.1. Participants’ characteristics

The background characteristics of the participants are summarized in Table 1. About one-sixth (16.8%) of the participants self-reported having tested positive either by NAAT or RAT for COVID-19.

**Table 1:** Participants’ characteristics.

	*n*	%
Socio-demographics		
Sex		
Female	335	67.0
Male	165	33.0
Age groups (years)		
18–30	71	14.2
31–60	262	52.4
>60	167	33.4
Educational level		
Below college	365	73.0
College or above	123	24.6
Missing data	12	2.4
Marital status		
Others	151	30.2
Married	344	68.8
Missing data	5	1.0
Employment status		
Full-time	208	41.6
Part-time	41	8.2
Retired	114	22.8
Under-employed	27	5.4
Homemaker	94	18.8
Others	16	3.2
Chronic disease status		
No/unknown	321	64.2
Yes	179	35.8
COVID-19 ever infection status		
No/Do not know/Refused to answer	416	83.2
Yes	84	16.8
Support toward the LWV policy		
No	302	60.4
Yes	198	39.6
Anticipated panic under the LWV policy		
No	379	75.8
Yes	121	24.2

LWV, living with the virus.

The mean (SD) scores of the subscales of the B-IPQ-COVID-19 (which ranged from 0 to 10), in descending order, were 6.3 (2.4) for consequences, 6.10 (2.0) for treatment control, 6.08 (1.8) for personal control, 5.8 (1.8) for illness coherence, 4.9 (1.9) for identity, 4.8 (2.6) for illness concern, 4.7 (1.9) for timeline, and 4.5 (2.7) for emotional representation. Between-group analyses found that (1) females reported higher levels of timeline (Cohen’s *d* = 0.27) and identity (Cohen’s *d* = 0.20) than males, (2) those of older age had higher levels of timeline (eta squared = 0.023) and emotional representations (eta squared = 0.017) and lower levels of personal control (eta squared = 0.012) and illness coherence (eta squared = 0.024), (3) those having educational level of college or above were more likely than others to have lower levels of consequences (Cohen’s *d* = 0.20), timeline (Cohen’s *d* = 0.37), and illness concern (Cohen’s *d* = 0.0.25) and a higher level of illness coherence (Cohen’s *d* = 0.22), and (4) those having chronic disease had higher levels of timeline (Cohen’s *d* = 0.23), treatment control (Cohen’s *d* = 0.19), and emotional representations (Cohen’s *d* = 0.19) and a lower level of illness coherence (Cohen’s *d* = 0.29) (see Supplementary Table S1).

### 3.2. Responses to the LWV policy

Of all the participants, 39.6% were supportive or strongly supportive of the LWV policy, while 24.2% stated that they would feel panicked under the LWV policy (Table 1). Anticipated panic under the LWV policy was negatively associated with the support toward the LWV policy (Pearson correlation coefficient = −0.56; *p* < 0.001).

### 3.3. Background factors of the responses to the LWV policy

In Table 2, those who were older, currently married, and not employed full-time (e.g., part-time job, retirement, and homemakers) were less likely than others to support the LWV policy and more likely than others to anticipate panic under the LWV policy. Those having an educational level of college or above were more likely than others to support the LWV policy and less likely to anticipate panic under the LWV policy. Chronic disease status was significantly and positively associated with anticipated panic but was not significantly associated with the support toward the LWV policy.

**Table 2:** Background factors of support and anticipated panic related to the LWV policy.

	DV = Support toward the LWV policy	DV = Anticipated panic under the LWV policy
	ORc (95% CI)	ORc (95% CI)
Sex		
Female	Reference = 1.0	Reference = 1.0
Male	1.38 (0.95, 2.02)	0.82 (0.53, 1.28)
Age group (years)		
18–30	Reference = 1.0	Reference = 1.0
31–60	0.50 (0.30, 0.85)*	1.51 (0.70, 3.24)
>60	0.38 (0.22, 0.67)**	4.39 (2.04, 9.44)***
Educational level		
Below college	Reference = 1.0	Reference = 1.0
College or above	2.24 (1.48, 3.40)***	0.46 (0.27, 0.80)**
Marital status		
Others	Reference = 1.0	Reference = 1.0
Married	0.64 (0.43, 0.94)*	1.81 (1.11, 2.93)*
Employment status		
Full-time	Reference = 1.0	Reference = 1.0
art-time	0.40 (0.19, 0.83)*	3.80 (1.76, 8.19)**
Retired	0.61 (0.37, 0.97)*	4.77 (2.72, 8.37)***
Under-employed	0.64 (0.28, 1.45)	1.27 (0.41, 3.99)
Homemaker	0.51 (0.30, 0.85)**	3.43 (1.88, 6.27)***
Others	0.65 (0.23, 1.85)	1.69 (0.45, 6.34)
Chronic disease status		
No/unknown	Reference = 1.0	Reference = 1.0
Yes	0.87 (0.60, 1.26)	2.05 (1.35, 3.12)**

DV, dependent variable; LWV, living with virus; ORc, crude odds ratio; CI, confidence interval. *p < 0.05; **p < 0.01; ***p < 0.001.

### 3.4. Associations between illness representations of COVID-19 and responses to the LWV policy

The multivariable logistic regression analyses showed that, after adjusting for the background factors, lower levels of consequences, timeline, identity, illness concern, and emotional representations were significantly associated with stronger support toward the LWV policy (the individual ORa ranged from 0.81 to 0.86). Consequences, timeline, identity, illness concern, and emotional representations were positively associated with anticipated panic under the LWV policy (the individual ORa ranged from 1.31 to 1.58). In addition, those having a higher level of illness coherence were more likely than others to support the LWV policy (ORa = 1.24) but the variable’s association with anticipated panic was statistically non-significant. Personal control and treatment control were not significantly associated with both support toward the LWV policy and anticipated panic under the LWV policy (Table 3).

**Table 3:** Associations between the illness representations of COVID-19 and support/anticipated panic related to the LWV policy.

	DV = Support toward the LWV policy	DV = Anticipated panic under the LWV policy
	ORa (95% CI)	ORa (95% CI)
Consequences	0.85 (0.78, 0.92)***	1.31 (1.18, 1.45)***
Timeline	0.86 (0.77, 0.95)**	1.38 (1.22, 1.56)***
Personal control	1.07 (0.96, 1.20)	0.95 (0.84, 1.07)
Treatment control	1.00 (0.91, 1.10)	0.97 (0.87, 1.07)
Identity	0.88 (0.80, 0.98)*	1.34 (1.18, 1.51)***
Illness concern	0.81 (0.75, 0.88)***	1.49 (1.34, 1.65)***
Illness coherence	1.24 (1.10, 1.39)***	0.99 (0.88, 1.11)
Emotional representations	0.86 (0.80, 0.93)***	1.58 (1.42, 1.76)***

DV, dependent variable; LWV, living with the virus; SD, standard deviation; ORa, adjusted odds ratio; CI, confidence interval. *p < 0.05; **p < 0.01; ***p < 0.001. The adjusted models were adjusted for background factors, including sex, age, educational level, marital status, chronic disease status, and employment status.

### 3.5. Moderation analyses

In Table 4, previous COVID-19 infection did not significantly moderate the associations between any of the eight illness representation domains and the support/anticipated panic related to the LWV policy.

**Table 4:** An analysis of the moderation effect of COVID-19 ever infection status for the associations between the illness representations of COVID-19 and the two dependent variables.

	DV = Support toward the LWV policy		DV = Anticipated panic due to the LWV policy	
	Main-effect-only models	Main-effect + Interaction term	Main-effect-only models	Main-effect + Interaction term
	ORa (95% CI)	ORa (95% CI)	ORa (95% CI)	ORa (95% CI)
	Model 1a	Model 2a	Model 3a	Model 4a
Consequence	0.84 (0.77, 0.92)***	0.83 (0.76, 0.92)***	1.33 (1.20, 1.48)***	1.35 (1.20, 1.52)***
COVID-19 ever infection	2.33 (1.38, 3.95)**	1.69 (0.36, 7.79)	0.30 (0.14, 0.66)**	0.73 (0.06, 8.83)
Consequence × COVID-19 ever infection		1.05 (0.84, 1.32)		0.89 (0.64, 1.23)
Δ − 2LL	0.195		0.487	
	Model 1b	Model 2b	Model 3b	Model 4b
Timeline	0.86 (0.78, 0.96)**	0.85 (0.76, 0.96)**	1.37 (1.21, 1.56)***	1.34 (1.17, 1.53)***
COVID-19 ever infection	2.10 (1.25, 3.53)**	1.43 (0.39, 5.28)	0.40 (0.19, 0.84)*	0.13 (0.01, 1.34)
Timeline × COVID-19 ever infection		1.09 (0.84, 1.42)		1.23 (0.83, 1.83)
Δ − 2LL	0.387		1.102	
	Model 1c	Model 2c	Model 3c	Model 4c
Personal control	1.06 (0.95, 1.18)	1.08 (0.96, 1.22)	0.97 (0.85, 1.09)	0.94 (0.83, 1.08)
COVID-19 ever infection	2.09 (1.25, 3.51)**	4.44 (0.74, 26.71)	0.38 (0.18, 0.79)*	0.13 (0.01, 1.64)
Personal control × COVID-19 ever infection		0.89 (0.68, 1.16)		1.18 (0.82, 1.70)
Δ − 2LL	0.742		0.827	
	Model 1d	Model 2d	Model 3d	Model 4d
Treatment control	1.01 (0.92, 1.11)	1.05 (0.94, 1.17)	0.95 (0.85, 1.05)	0.95 (0.84, 1.07)
COVID-19 ever infection	2.16 (1.29, 3.62)**	4.97 (1.15, 21.52)*	0.36 (0.17, 0.75)**	0.38 (0.07, 2.10)
Treatment control × COVID-19 ever infection		0.87 (0.69, 1.10)		0.99 (0.74, 1.32)
Δ − 2LL	1.443		0.006	
	Model 1e	Model 2e	Model 3e	Model 4e
Identity	0.89 (0.81, 0.99)*	0.86 (0.76, 0.97)*	1.32 (1.17, 1.50)***	1.33 (1.16, 1.51)***
COVID-19 ever infection	2.05 (1.23, 3.44)**	0.90 (0.26, 3.15)	0.43 (0.20, 0.90)*	0.49 (0.07, 3.47)
Identity × COVID-19 ever infection		1.20 (0.93, 1.55)		0.97 (0.68, 1.39)
Δ − 2LL	1.983		0.025	
	Model 1f	Model 2f	Model 3f	Model 4f
Illness concern	0.81 (0.75, 0.88)***	0.81 (0.74, 0.89)***	1.49 (1.34, 1.66)***	1.50 (1.34, 1.68)***
COVID-19 ever infection	1.99 (1.17, 3.37)*	1.88 (0.69, 5.13)	0.37 (0.16, 0.84)*	0.44 (0.06, 3.10)
Illness concern × COVID-19 ever infection		1.01 (0.83, 1.23)		0.97 (0.73, 1.30)
Δ − 2LL	0.018		0.040	
	Model 1g	Model 2g	Model 3g	Model 4g
Illness coherence	1.22 (1.09, 1.37)**	1.24 (1.08, 1.41)**	1.00 (0.89, 1.13)	1.01 (0.89, 1.15)
COVID-19 ever infection	2.00 (1.19, 3.36)**	2.69 (0.44, 16.54)	0.37 (0.18, 0.78)**	0.67 (0.08, 5.74)
Illness coherence × COVID-19 ever infection		0.95 (0.72, 1.27)		0.91 (0.64, 1.29)
Δ − 2LL	0.110		0.299	
	Model 1h	Model 2h	Model 3h	Model 4h
Emotional representations	0.87 (0.80, 0.93)***	0.85 (0.78, 0.92)***	1.58 (1.42, 1.77)***	1.58 (1.41, 1.78)***
COVID-19 ever infection	2.07 (1.23, 3.48)**	1.35 (0.53, 3.43)	0.33 (0.14, 0.76)**	0.33 (0.04, 2.86)
Emotional representations × COVID-19 ever infection		1.11 (0.92, 1.33)		1.00 (0.73, 1.37)
Δ − 2LL	1.153		<0.001	

DV, dependent variable; LWV, living with the virus; ORa, adjusted odds ratio; CI, confidence interval; LL, log likelihood; *p < 0.05; **p < 0.01; ***p < 0.001. The models were adjusted for background factors, including sex, age, educational level, marital status, occupation, and chronic disease status.

## 4. Discussion

About three-fifths of the participants supported the LWV policy at the time of the survey when the zero-COVID-19 policy but not the LWV policy was adopted in Hong Kong. As there was then an ongoing severe COVID-19 outbreak in Hong Kong that caused over 9,000 deaths, it is understandable that a sizable proportion of the public preferred not to adopt the LWV policy which would lift the strict control measures and increase the risk of transmission. In contrast, even in the presence of such a severe outbreak, about 40% of the participants still supported the removal of the key control measures, plausibly because many people felt tired of living under the restrictive control conditions (26). Prevention fatigue is common (27). The international trends of lifting the key COVID-19 control measures may further boost support toward the LWV policy in Hong Kong in the near future. To inform policymakers, it is warranted to understand whether similar divisive views are present in countries having and having not adopted the LWV policy, especially in developing countries, and trace how such views would change over time. Such data is not available and a knowledge gap exists.

As of May 12, 2022, the 2-dose vaccination rate in Hong Kong was 85% (over 90% for one dose) (28). It was estimated that over 60% of the population had been infected with COVID-19 (28) and more infections occurred since then. With immunity building up in the community, COVID-19 is possibly becoming an endemic in Hong Kong. During the study period (i.e., March to April 2022), the Hong Kong government officially and strictly adopted the zero-COVID-19 policy (29) but has loosened some control policies since May 2022 (e.g., reopening entertainment venues and schools and allowing non-residents to enter Hong Kong). The city may be gradually moving toward adopting the LWV policy. It is hence warranted to increase the support toward the LWV policy, which is a required condition for the effective implementation of the LWV policy.

In general, support toward governmental COVID-19 control policies was negatively associated with mental distress in the general population (30) which had been elevated during the pandemic (31, 32). Importantly, about a quarter of the public anticipated panic under the LWV policy. This potential unintended consequence of the LWV policy may have been overlooked by researchers and policymakers. Meta-analyses have alerted stakeholders that the pandemic had caused excessive depression globally (33, 34); the prevalence of depression also increased during the pandemic in Hong Kong (35) due to resource losses (36), quarantines (37), and social distancing (38, 39). Anticipated panic is understandable given the ongoing severe outbreak in Hong Kong during the survey period. Hence, mental health promotion and reassurance need to accompany the shift toward the LWV policy. To reduce panic due to the LWV policy, the government should protect and support vulnerable groups (e.g., people who were older, less educated, and have chronic diseases). For instance, comorbidity with chronic disease(s) would greatly increase the risk of COVID-19-related deaths (40), while such patients were less likely than others to have taken up COVID-19 vaccination (41) and have sub-optimal access to updated information related to COVID-19 (42). Although the LWV policy might amplify the heavy burden of mental distress, interestingly, it may ameliorate mental distress by reducing some stressors (e.g., social distancing and unemployment) at a population level (43). Future studies should investigate the net impact of the LWV policy on the population mental distress.

This study provides updated information about how people think and feel about COVID-19 (i.e., illness representations of COVID-19). The observed mean scores of the B-IPQ-COVID-19 constructs can be compared to those of a previous local population-based study conducted in Hong Kong in April 2020 (9) that used the same methodology. The levels have decreased over time in the domains of consequence (8.3 versus 6.3), timeline (6.8 versus 4.7), identity (7.0 versus 4.9), illness concern (6.8 versus 4.8), emotional representations (5.5 versus 4.5), and treatment control (6.4 versus 6.1); the level of illness coherence (5.9 versus 5.8) remained similar. Thus, over the last 2 years, the general public in Hong Kong found COVID-19 less consequential, less chronic, and having fewer symptoms, plausibly due to the milder symptoms and shorter illness duration caused by the Omicron variants (44). As a result, concerns and negative emotional representations may have declined over time (3). The identity domain has special relevance, as a zero score implies that an individual believes that he/she would be asymptomatic while a score of 10 implies that he/she would have many severe COVID-19 symptoms in case of infection. The decline in the identity score suggests that more people believe that COVID-19 infection does not involve many symptoms. Treatment control declined slightly over time. Approval of Paxlovid and Molnupiravir was announced in March 2022 and was first used in Hong Kong in April 2022. Most of the participants might not know about the medicines as the survey was conducted from March 7th to April 19th, 2022. Personal control increased over time (5.1 versus 6.1), plausibly because many COVID-19 infections were mild and self-limiting and did not need clinical support. An important message is that cognitions and emotions related to COVID-19 are dynamic and surveillance is warranted. In addition, between-group comparisons of illness representation scores by some background factors in this study further identify high-risk subgroups that needed tailored health promotion to improve specific illness representations (e.g., lower level of illness coherence in the age group of >60 years).

The present study found significant associations between some illness representations and support/anticipated panic regarding the LWV policy. According to the CSM, the cognitive and emotional illness representations would affect coping, which would affect disease-related outcomes (policy support and anticipated panic in this case) (6). Higher levels of the cognitive constructs of consequences, timeline, and identity were significantly associated with lower supportiveness, which implies that COVID-19 control measures are perceived to be less important. Significant negative associations between these illness representations and anticipated panic under the LWV policy are observed. Such findings are intuitive as people possessing such perceptions might worry less about the removal of control measures under the LWV policy. When implementing the LWV policy, the government needs to explain to the public that the Omicron variants often result in milder or no symptoms and have relatively short symptomatic periods. Furthermore, illness concern and emotional representations were negatively associated with the support toward the LWV policy and positively associated with anticipated panic. In the literature, support toward governmental policy was negatively associated with emotional responses to COVID-19 (30). Stakeholders need to be sensitized about potential emotional responses to the LWV policy which has not been studied in the literature.

Illness coherence (people’s understanding of COVID-19) was significantly associated with policy support but not with anticipated panic in this study. To increase support toward lifting the control measures when transiting into the endemic phase of COVID-19, the general public’s knowledge about COVID-19 needs to be enhanced and updated regularly, especially among older and less educated people. It is unexpected that treatment control was not significantly associated with the two types of responses. As mentioned, the public may not know much about the newly available medicines. Future studies should evaluate whether the provision of knowledge about the effectiveness of the medicines would affect the public’s support and anticipated panic related to the LWV policy. This study found that the associations between the illness representations of COVID-19 and support/anticipated panic regarding the LWV policy were not moderated by previous COVID-19 infection. Thus, the illness representation framework should be applicable to understand responses to the LWV policy in both the infected and uninfected groups.

Cautions about the contextual situations in Hong Kong should be made when generalizing the findings of this study to other countries. For instance, Hong Kong was affected by a socio-political movement from June to December 2019, which had calmed down since January 2020. The movement was about confrontation with the government and hence might have lowered the trust toward the government; the lower level of trust toward the government might affect supportiveness toward any governmental policies (including both the zero-COVID-19 policy and the LWV policy). In addition, the government and the public were keen to resume normal traveling (i.e., no quarantine) from Hong Kong to mainland China, while the zero-COVID-19 policy was a potential requirement for lifting the restrictions; some people might not support the LWV policy as it would mean more infections and hindrance against the removal of the Hong Kong/mainland travel restriction. Despite these cautions, some generalizations may still be relevant and feasible. First, the research question is a general one, as more and more societies are gradually moving toward the LWV policy while the supportiveness toward the policy in the general population would not be unanimous and negative emotional responses are expected because of the higher risk of infection upon removal of the control measures. Second, the research framework is general. People of all societies would develop illness representations of COVID-19. It is expected that the association between illness representations and supportiveness and emotions related to the LWV policy would exist across societies, although the levels of the studied variables and the strengths of associations might empirically vary. Future international comparative studies are warranted.

To recapitulate, the findings have significant international implications. Most countries have implemented some COVID-19 control measures. As all of them may need to remove the control measures gradually to let people live normally and seek economic recovery, issues concerning the LWV policy are globally relevant. Policymakers would certainly like to understand the levels of support and anticipated panic related to the LWV policy as well as how people think and feel about the pandemic. The key findings of this study about the split in opinions and the presence of prevalent anticipated panic would alert policymakers, especially those of developing countries that are less prepared to adopt the LWV policy, about some foreseeable issues. The findings regarding the identified factors of the LWV policy would offer insights for health promotion to improve supportiveness and reduce panic due to the LWV policy. This study also poses a new research question to understand the ending phase of the COVID-19 pandemic and future pandemics involving stringent control policies and extends the applications of the construct of illness representations.

This study has some limitations. First, although the response rate was comparable to other local telephone surveys (45, 46), it was only 52.2%; the respondents and non-respondents might have different characteristics. Although the age distribution of the population in this study was comparable to that of the 2021 Hong Kong census data, females were over-represented. Sex was, however, not associated with the two dependent variables in this study. Third, social desirability bias may exist. As the zero-COVID-19 policy was the official strategy of the mainland Chinese and Hong Kong governments, it may be considered socially desirable to be unsupportive of the LWV policy, which may underestimate the level of supportiveness. Fourth, due to the cross-sectional design, causal or temporal inference is precluded; the findings should be confirmed by longitudinal studies. Last, the scales assessing the two dependent variables were constructed for this study as no validated tools existed in the literature; future studies are needed to confirm their reliability and validity.

In conclusion, this study observed that about 40% of participants supported the LWV policy and about 25% anticipated panic under the LWV policy. Policy support was negatively associated with anticipated panic due to the LWV policy. Over the last 2 years, COVID-19 was perceived to be less severe, have fewer negative emotions, and be more controllable by personal acts; it also induced fewer emotional representations. Irrespective of previous COVID-19 infection status, most of the illness representations were significantly associated with policy support and anticipated panic. Better policy support and less panic might occur due to the changes in illness representations of COVID-19. Governments, especially those of countries with lower preparedness, might prepare for transitions to the LWV policy and be alerted that it might threaten some people. To approach consensus and alleviate potential panic, preparedness such as boosting the vaccination rate and enhancing health communication and support to vulnerable groups are required. The present findings provide good grounds for changing perceptions of COVID-19 in general populations. Stepwise relaxation of the control measures, instead of overnight changes may be considered.

## Data availability statement

The raw data supporting the conclusions of this article will be made available by the authors, without undue reservation.

## Ethics statement

The studies involving humans were approved by the Survey and Behavioral Research Ethics Committee of the Chinese University of Hong Kong (No. SBRE-21-0555A). The studies were conducted in accordance with the local legislation and institutional requirements. The ethics committee/institutional review board waived the requirement of written informed consent for participation from the participants or the participants’ legal guardians/next of kin because verbal informed consent is acceptable in telephone surveys.

## Author contributions

JL and YY: conceptualization. YY and JL: methodology and writing – original draft. RL, JN, TI, and ML: investigation. YY: software and formal analysis. YY, ML, and JL: data curation. JL: validation, resources, supervision, and funding acquisition. YY, RL, JN, and JL: writing – review and editing. All authors contributed to the article and approved the submitted version.

## Funding

This study was supported by internal research funding of the Centre for Health Behaviour Research of the Chinese University of Hong Kong. The funding source has no role in this study.

## Conflict of interest

The authors declare that the research was conducted in the absence of any commercial or financial relationships that could be construed as a potential conflict of interest.

## Publisher’s note

All claims expressed in this article are solely those of the authors and do not necessarily represent those of their affiliated organizations, or those of the publisher, the editors and the reviewers. Any product that may be evaluated in this article, or claim that may be made by its manufacturer, is not guaranteed or endorsed by the publisher.
