# Supplementary material for: Illness representation of COVID-19 affected public’s support and anticipated panic regarding the living with the virus policy: a cross-sectional study in a Chinese general population
Source: Front Public Health. 2023 Sep 4;11:1158096. doi: 10.3389/fpubh.2023.1158096 (PMC10506401; doi:10.3389/fpubh.2023.1158096)
Supplement: Supplementary file 1 [file Table_1.DOCX]

Supplementary Material

Table S1 Between-group comparisons of illness representation scores by background factors

|  | Sex | | | | Age group (years) | | | | | Educational level | | | | Chronic disease status | | | |
| --- | --- | --- | --- | --- | --- | --- | --- | --- | --- | --- | --- | --- | --- | --- | --- | --- | --- |
|  | Female | Male | *p* | Cohen’s *d* | 18-30 | 31-60 | >60 | *p* | Eta squared | Below college | College or above | *p* | Cohen’s *d* | No/ unknown | Yes | *p* | Cohen’s *d* |
|  | Mean (SD) | Mean (SD) |  |  | Mean (SD) | Mean (SD) | Mean (SD) |  |  | Mean (SD) | Mean (SD) |  |  | Mean (SD) | Mean (SD) |  |  |
| Consequences | 6.3 (2.3) | 6.1 (2.4) | 0.301 | 0.10 | 5.8 (2.4) | 6.3 (2.2) | 6.3 (2.5) | 0.249 | 0.006 | 6.4 (2.3) | 5.9 (2.5) | 0.046 | 0.20 | 6.2 (2.3) | 6.3 (2.4) | 0.723 | 0.03 |
|  |  |  |  |  |  |  |  |  |  |  |  |  |  |  |  |  |  |
| Timeline | 4.9 (2.0) | 4.4 (1.8) | 0.006 | 0.27 | 4.0 (1.8) | 4.8 (1.8) | 5.0 (2.0) | 0.003 | 0.023 | 4.9 (1.9) | 4.2 (1.8) | <0.001 | 0.37 | 4.6 (1.9) | 5.0 (2.0) | 0.013 | 0.23 |
|  |  |  |  |  |  |  |  |  |  |  |  |  |  |  |  |  |  |
| Personal control | 6.0 (1.8) | 6.2 (1.9) | 0.535 | 0.06 | 6.1 (1.7) | 6.2 (1.6) | 5.8 (2.1) | 0.046 | 0.012 | 6.1 (1.8) | 6.0 (1.9) | 0.750 | 0.03 | 6.1 (1.8) | 6.0 (1.9) | 0.294 | 0.09 |
|  |  |  |  |  |  |  |  |  |  |  |  |  |  |  |  |  |  |
| Treatment control | 6.1 (2.0) | 6.2 (2.1) | 0.690 | 0.04 | 6.0 (2.0) | 6.0 (1.8) | 6.3 (2.3) | 0.470 | 0.003 | 6.2 (2.0) | 5.9 (2.3) | 0.262 | 0.12 | 6.0 (2.0) | 6.4 (2.0) | 0.042 | 0.19 |
|  |  |  |  |  |  |  |  |  |  |  |  |  |  |  |  |  |  |
| Identity | 5.0 (1.9) | 4.6 (1.9) | 0.036 | 0.20 | 4.8 (2.1) | 4.9 (1.8) | 5.0 (2.0) | 0.722 | 0.001 | 4.9 (1.9) | 4.7 (1.9) | 0.337 | 0.10 | 4.8 (1.8) | 5.0 (2.0) | 0.410 | 0.07 |
|  |  |  |  |  |  |  |  |  |  |  |  |  |  |  |  |  |  |
| Illness concern | 5.0 (2.6) | 4.5 (2.6) | 0.052 | 0.18 | 4.5 (2.4) | 4.9 (2.4) | 4.9 (2.9) | 0.528 | 0.003 | 5.0 (2.7) | 4.3 (2.4) | 0.019 | 0.25 | 4.8 (2.6) | 5.0 (2.7) | 0.450 | 0.07 |
|  |  |  |  |  |  |  |  |  |  |  |  |  |  |  |  |  |  |
| Illness coherence | 5.7 (1.9) | 5.9 (1.8) | 0.470 | 0.07 | 5.8 (1.8) | 6.0 (1.5) | 5.4 (2.2) | 0.002 | 0.024 | 5.7 (1.8) | 6.1 (1.8) | 0.042 | 0.22 | 6.0 (1.7) | 5.4 (2.1) | 0.003 | 0.29 |
|  |  |  |  |  |  |  |  |  |  |  |  |  |  |  |  |  |  |
| Emotional representations | 4.6 (2.7) | 4.2 (2.6) | 0.127 | 0.15 | 4.1 (2.3) | 4.3 (2.6) | 5.0 (2.9) | 0.016 | 0.017 | 4.6 (2.8) | 4.2 (2.5) | 0.138 | 0.16 | 4.3 (2.6) | 4.8 (2.8) | 0.036 | 0.19 |

Note. Cohen’s *d* values of 0.20, 0.50, and 0.80 indicate small, medium, and large effect size, respectively. Eta squared values of 0.010, 0.059, and 0.138 indicate small, medium, and large effect size, respectively.
